# An application of the stress-diathesis model: A review about the association between smoking tobacco, smoking cessation, and mental health

**DOI:** 10.1016/j.ijchp.2022.100335

**Published:** 2022-10-01

**Authors:** Gemma M.J. Taylor, Jorien L. Treur

**Affiliations:** a Department of Psychology, University of Bath, 10 West, Bath BA2 7AY, United Kingdom; b Department of Psychiatry, University of Amsterdam, Amsterdam UMC, Amsterdam, the Netherlands

**Keywords:** Smoking cessation, Mental health, Tobacco, Addiction, Stress-diathesis, Epidemiology, Public health

## Abstract

**Background:**

Worldwide, approximately 24% of all adults smoke, but smoking is up to twice as prevalent in people with mental ill-health. There is growing evidence that smoking may be a causal risk factor in the development of mental illness, and that smoking cessation leads to improved mental health.

**Methods:**

In this scholarly review we have: (1) used a modern adaptation of the Bradford-Hill criteria to bolster the argument that smoking could cause mental ill-health and that smoking cessation could reverse these effects, and (2) by considering psychological, biological, and environmental factors, we have structured the evidence to-date into a stress-diathesis model.

**Results:**

Our model suggests that smoking is a psychobiological stressor, but that the magnitude of this effect is mediated and modulated by the individual's diathesis to develop mental ill-health and other vulnerability and protective factors. We explore biological mechanisms that underpin the model, such as tobacco induced damage to neurological systems and oxidative stress pathways. Furthermore, we discuss evidence indicating that it is likely that these systems repair after smoking cessation, leading to better mental health.

**Conclusion:**

Based on a large body of literature including experimental, observational, and novel causal inference studies, there is consistent evidence showing that smoking can negatively affect the brain and mental health, and that smoking cessation could reverse the mental ill-health caused by smoking. Our model suggests that smoking prevention and treatment strategies have a role in preventing and treating mental illness as well as physical illness.

## Background

Smoking tobacco is the world's leading cause of preventable disease and death (Cancer Research UK, 2020; World Health Organisation, 2011). The global healthcare expenditure due to smoking-attributable disease is estimated at $467 billion with the economic burden being most prominent in Europe and North America (Goodchild et al., 2022). Worldwide, approximately 24% of all adults smoke (The World Bank, 2018). In high income countries like the UK, smoking prevalence has decreased from 29% during the 1990s to about 15% in recent years (Reitsma et al., 2017). However, smoking is twice as prevalent in people with mental ill-health (Taylor et al., 2019). In the UK, 34% of people with depression, 29% of people with anxiety and 44% of people with severe mental ill-health like schizophrenia smoke tobacco (Taylor et al., 2019); patterns which are replicated in, for example, the USA, the Netherlands, and Australia (Centers for Disease Control and Prevention (CDC), 2011; Lawrence et al., 2013; Vermeulen-Smit et al., 2015). People with mental ill-health have a 19% reduction in the odds of achieving abstinence when attempting to stop (Hitsman et al., 2013), but are as motivated to stop as those without mental ill-health (Siru et al., 2009). These differences increase mortality in people with mental ill-health when compared to the general population resulting from cancer (mortality rate ratio: 1·92 [95% confidence interval: 1·91–1·94]) (Plana-Ripoll et al., 2019), cardiovascular disease (mortality hazard ratio: 1·85 [95% CI: 1·53–2·24]) and other illness (Correll et al., 2017).

The nature of the association between smoking and mental ill-health has been debated over the years. One likely explanation for the co-occurrence of smoking and mental ill-health is that they share underlying risk factors. These shared risk factors could be environmental (e.g., socio-economic) and/or genetic in origin (Hiscock et al., 2012; Royal College of Physicians and Royal College of Psychiatrists, 2013). The hypothesis that smoking could have a negative (causal) effect on mental health has remained elusive. There are plausible biological mechanisms that could explain the harmful effects of tobacco – such as neuroadaptations in nicotinic pathways in the brain, inflammation, and oxidative stress – but because smoking and mental health are both complex and multifactorial traits, causal inference using traditional epidemiological approaches has been extremely challenging.

A recent Cochrane systematic review by Taylor and colleagues found consistent evidence that smoking cessation is linked to later improvements in mental health, with an effect size equal to antidepressant treatment (Taylor et al., 2021, 2014, 2020a). Yet, many smokers and health care providers report that smoking offers mental health benefits. Specifically, smokers claim that smoking helps with their mood, symptoms of stress, and can act as a form of therapeutic self-harm (Taylor et al., 2020b). A systematic review of 16,369 mental health professionals’ attitudes to treating tobacco addiction in people with mental ill-health found, similar to other studies in this area, that negative attitudes towards smoking cessation are widespread (Huddlestone et al., 2018; Sheals et al., 2016; Smith et al., 2019). Clinicians report that smoking helps patients with their mental ill-health symptoms, without smoking patients could not cope, and helping patients to stop smoking would be taking away one of their few pleasures in = life and they will be harming their patients' mental health (Huddlestone et al., 2018; Sheals et al., 2016; Smith et al., 2019). These misunderstandings perpetuate the failure to treat tobacco addiction in mental health settings and contribute to continuing high smoking rates in this population.

The idea that smoking alleviates symptoms of mental ill-health is known as the self-medication hypothesis, and is psychodynamic in origin (Khantzian, 1997). There is evidence from randomised experiments in humans that nicotine leads to short-term benefits in memory, attention and other cognitive functions (Dondé et al., 2020; Foulds et al., 1996), and this is further supported by animal and neurobiological models (Alhowail, 2021; Counotte et al., 2012). These short-term effects of nicotine appear to be particularly important in reducing negative symptoms in schizophrenia (Levin et al., 2005), and have some salience amongst people with attention deficit hyperactivity disorder (Kollins et al., 2020). There is also evidence that nicotine can lead to release of neurotransmitters resulting in feeling reward and pleasure (Dani & De Biasi, 2001; Mansvelder & McGehee, 2002; Nestler, 2005). However, nicotine withdrawal is also associated with increases in reward thresholds (Epping-Jordan et al., 1998) suggesting that these effects are likely to initiate, rather than maintain addiction. It is possible though that smokers with mental ill-health are more susceptible to tobacco dependency because of these effects.

We argue that if the self-medication hypothesis was applicable, smoking would offer measurable psychological benefits, and that symptoms of mental ill-health would improve after starting to smoke. Yet, there is strong longitudinal evidence showing that the opposite is true: smoking is associated with an increase in mental health problems and smoking cessation is associated with mental health benefits. This discrepancy (between the feeling that smoking offers mental health benefits and the opposite being observed in research studies) can be explained by the misattribution hypothesis, which suggests that smokers mistake the ability of smoking to relieve tobacco-withdrawal symptoms for its ability to alleviate symptoms of mental ill-health. We present more evidence on this hypothesis, including potential biological pathways further on in our manuscript.

In this narrative review we will:

(a) use a modern adaptation of the Bradford-Hill criteria to bolster the argument that smoking causes mental ill-health and that smoking cessation could reverse the mental ill-health caused by smoking, and
(b) propose a new model for the causal association between smoking, smoking cessation, and mental health.

## The association between smoking, smoking cessation, and mental health: the evidence presented according to the Bradford-Hill criteria for causal inference

Observational studies that have used methods permitting causal inference (e.g., instrumental variable methods or properly adjusted longitudinal studies), indicate that smoking is a likely causal risk factor in the development of depression, schizophrenia, and bipolar disorder (Taylor et al., 2019; Treur et al., 2021; Vermeulen et al., 2019) and that smoking cessation leads to a reduction in the prescription of psychoactive medications like antidepressants and anxiolytics. The Bradford-Hill criteria can be used to weigh up evidence when making causal inferences from epidemiological studies. There are various modern adaptations of Bradford-Hill's criteria (Hill, 1965; Howick et al., 2009; Schünemann et al., 2011; Shimonovich et al., 2020), but in essence these all generally agree that to assess causality the association in question should be measured up against the following criteria: strength and consistency of the association, the role of confounding, temporality/reverse causation, study design suitability, and biological plausibility. In this paper we predominately use the Shimonovich version of the Bradford-Hill criteria, which is an adaptation of the GRADE (Grading of Recommendations, Assessment, Development and Evaluations) criteria (Schünemann et al., 2011) to assess the causal association between smoking, smoking cessation, and mental health (Shimonovich et al., 2020).

### Strength and consistency

A systematic review and meta-analysis of 102 studies examined the association between change in mental health after smoking cessation, compared to continued smoking (Taylor et al., 2021, 2014). The review included longitudinal studies of any clinical population, that reported change in anxiety, depression, positive affect, psychological quality of life, or stress from pre-quit attempt to follow-up of 6-weeks or longer (i.e., after the withdrawal period). The meta-analysis consistently showed that people who stopped smoking, on average, experienced an improvement across all mental health outcomes. The association was robust to multiple sensitivity analyses examining the potential impact of study- or population-level risks of bias. The strength of the pooled standardised mean difference in change was clinically important - the association between smoking cessation and change in depression and anxiety was equal to or larger than the estimate from trials of antidepressants versus placebo. A meta-analysis of individual-level data from randomised controlled trials (RCTs) of antidepressants for "mild" to "severe" depression found that antidepressants resulted in improved depression symptoms for people with “mild” depression, standardised mean difference (SMD) (−0·11 [95% CI: −0·26 to −0·04]), and improved depression symptoms in people with “severe” depression (SMD −0·47 [95% CI: −0·59 to −0·34]) (Fournier et al., 2010). In the Cochrane review the effect size for smoking cessation and depression was, SMD −0·30 [95% CI: −0·39 to −0·21], which falls within this range. A meta-analysis of 34 RCTs examined the effect of antidepressants on generalised anxiety disorder, and effect estimates ranged from SMD −0·23 [95% CI: −0·32 to −0·14] to SMD −0·50 [95% CI: −0·77 to −0·23] (National Collaborating Centre for Mental Health, 2011); this was similar to the effect size for smoking cessation and anxiety, SMD −0·28 [95% CI: −0·43 to −0·13]. A systematic review conducted by Fluharty et al. (2017) which included longitudinal cohort studies, examined the impact of smoking on depression and anxiety. Over a third of studies found that smoking at baseline led to later onset of anxiety or depression. Importantly, there were no studies showing that smoking was associated with a later reduction in anxiety or depression rates.

### Confounding

In the meta-analysis conducted by Taylor et al. (2021) some studies supplied adjusted and unadjusted estimates for the association between smoking cessation and change in mental health. The covariates included, amongst others: age, sex, education, working status, smoking and nicotine dependency, mental health status, report of calming effects from smoking, and receipt of smoking cessation medication. Comparison of these adjusted estimates with unadjusted estimates indicated no meaningful change in the results. Another study triangulated evidence for the relative effect of varenicline compared to nicotine replacement therapy on mental health outcomes (i.e., depression, anxiety, antidepressants, and anxiolytics) (Taylor et al., 2019). Varenicline is well-known to be more effective for smoking cessation than is single nicotine replacement therapy. Besides the impact of these medicines on smoking cessation, the authors additionally looked at the impact on mental health. Because varenicline is more effective, the varenicline arm comprised a higher percentage of individuals who had successfully stopped smoking. The study used three analytical approaches, each with different ability to control for residual confounding: multivariable regression modelling (little control), propensity score matched regression modelling (more control), and instrumental variable analysis (unlikely to be affected by confounding if certain underlying assumptions are met). All three models consistently found that varenicline was associated with reduced odds of receiving mental health diagnoses and prescriptions up to 2-years, suggesting that it was unlikely that confounding drives the association between smoking cessation and mental health benefits. It could be that the reduction in antidepressant and anxiolytic prescriptions was due to pharmacokinetic or pharmacodynamic interactions between smoking and these medicines. Smoking increases metabolism of some antipsychotics, necessitating higher doses (Royal College of Psychiatrists, 2010); thus, upon smoking cessation antipsychotic medicine dose needs to be reduced. However, to our knowledge there are no studies showing a pharmacokinetic or pharmacodynamic pathway that could lead to a reduction in antidepressant and anxiolytic medicine doses because of long-term smoking cessation. Finally, several studies have been conducted that used Mendelian randomisation. By employing genotype information as an instrumental variable, Mendelian randomisation provides better control for residual confounding. Because genetic risk variants are randomly assigned during meiosis, they should not be associated with confounders and can be used to ‘proxy’ smoking and test potential effects on mental health (Katikireddi et al., 2018). So far, Mendelian randomization studies have shown compelling evidence that smoking is a causal risk factor for depression, bipolar disorder, and schizophrenia (Treur et al., 2021). Given the strong premise of this method, confounding should not have affected these findings. Combined with the observational findings described above, this makes for robust evidence that smoking can cause mental ill-health, and that smoking cessation can improve mental health.

**Box 1:** Review summary.

***What is known on this topic?***
It is well-known that there is a strong association between smoking tobacco and mental ill-health, yet the causal nature of this association, and what happens after smoking cessation is not clear-cut.
***What this model adds?***
By considering psychological, biological, and environmental factors, we have structured the evidence to-date according to a stress-diathesis model. Our model suggests that smoking is a psychobiological stressor, but that the magnitude of this effect is mediated and modulated by the individuals’ diathesis to develop mental ill-health and other vulnerability and protective factors.
***What are the implications?***
Smoking tobacco damages the brain and mental health in the same way that smoking damages other organs and physical health.
**Smoking cessation could reverse the mental ill-health caused by smoking.**
On average, mental health will improve after stopping smoking and breaking the tobacco withdrawal cycle, and at minimum smoking cessation will not harm mental health.
Smoking cessation can be a treatment for mental illness, and smoking prevention strategies have a role in preventing mental illness as well as physical illness.
***Areas for future research to test and contribute towards this model?***
Identifying which traits mediate and modulate mental health outcomes in smoking cessation treatments.
Determining the predictive strength of this model in different types of mental health disorders.
Disentangling which constituents of tobacco smoke are to blame for mental ill-health, and the implications that this has for the role that e-cigarettes may play in mental health.
Examining the role of exposure duration on mental health, i.e., is there a dose-response effect of smoking cessation duration on mental health improvements?

### Temporality and study design suitability

It could be that the observed associations between smoking and mental ill-health are explained by reverse causation – for instance, that mental ill-health leads people to smoke more, or that improved mental health leads people to attempt cessation. Of the 102 studies included in the systematic review conducted by Taylor et al. (2021), 56 were secondary analyses of RCTs. In these trials most people attempted cessation, and therefore their decision to stop smoking was not dependant on change in mental health. The observed change in mental health was measured after entry into the trials. It could be that improved mental health predicts the likelihood of smoking cessation or that worsened mental health after smoking cessation predicts relapse to smoking. However, in a previous longitudinal analysis of RCT data there was no greater likelihood to relapse at 1-year for those whose mental health worsened after cessation compared with those whose mental health stayed the same or improved; odds ratio (OR) 1·01 [95% CI: 0·97 to 1·05] (Taylor et al., 2015). There was one study in which participants were randomised to stop or to continue smoking, with a short-term assessment of anxiety and depression symptoms at 3-months, which produced an effect estimate similar to that seen in the observational data (Dawkins et al., 2009). It should be noted though, that in this study differential attrition, notably from the arm assigned to stop smoking, impacts interpretation. Additional evidence that the association is not, or not merely, due to reverse causation comes from the Mendelian randomization studies conducted so far. For instance, there was strong and consistent evidence that smoking was a causal risk factor for bipolar disorder, but no evidence for the reverse (Vermeulen et al., 2019) For smoking in relation to depression and schizophrenia there was evidence for bi-directional causal effects, but the strength of evidence was markedly stronger that smoking leads to mental ill-health than the reverse, that mental ill-health leads to smoking (Treur et al., 2021).

### Biological plausibility

The theory that smoking cessation improves mental health is supported by a plausible biological mechanism, related to neuroadaptations in nicotinic pathways in the brain (Benowitz et al., 2009). Neuroadaptations in these pathways are associated with the occurrence of psychological withdrawal symptoms, such as depressed mood, agitation, and anxiety. Withdrawal symptoms are alleviated by smoking and remain alleviated shortly after smoking, but symptoms return when blood levels of nicotine decline at around 20-minutes after smoking (Benowitz et al., 2009). This withdrawal cycle is marked by repeated changes in a smoker's psychological state throughout the day (Benowitz, 2010; Benowitz et al., 2009; Parrott, 2003). Constant fluctuation in withdrawal-induced psychological symptoms experienced by smokers could worsen mental health overtime, and the associated biological effects could increase the risk of mental ill-health (Taylor & Munafò, 2019). Smokers can mistake the ability of tobacco to alleviate tobacco withdrawal for an ability to alleviate mental ill-health symptoms. After breaking the tobacco withdrawal cycle, through smoking cessation, these systems recover (Mamede et al., 2007), possibly in the same way that other systems damaged by smoking reverse after smoking cessation (Doll et al., 2004; Pirie et al., 2013). This is in line with evidence from ex-smokers who report that withdrawal symptoms abate a few weeks after smoking cessation (Hughes, 2007).

Another potential biological pathway is that smoking could lead to mental ill-health via inflammation and oxidative stress (Hallstrand et al., 2014; Salem et al., 2021). There is strong evidence that inflammation and oxidative stress underlie the progression of depression, anxiety and bipolar disorder (Berk et al., 2011; Milaneschi et al., 2021). At the same time, it is well established that smoking has long-term effects on the oxidative stress burden. In a cohort study of 3835 participants, Salem et al. (2021) found that current smoking was associated with increased oxidative stress biomarkers (i.e., total thiol groups of serum proteins, and derivatives of reactive oxygen metabolites). Crucially, the association showed a dose-response pattern, such that higher levels of daily smoking led to higher concentrations of biomarkers. While it has not yet been explicitly tested, it is plausible that negative effects of smoking on mental health are mediated and modulated by oxidative stress pathways. There is also evidence to support the notion that smoking cessation can reverse the damage caused by smoking - as those who had stopped smoking for more than 10 -years had similar oxidative stress biomarker levels as never smokers (Salem et al., 2021).

## Discussion: a new model for the association between smoking and mental health

Smoking tobacco is a psychological and biological stressor; it damages every organ in the body (World Health Organisation 2011) . It is therefore curious to argue that smoking selectively offers lasting mental health benefits, while simutaneously damaging physical health. To make a strong case for negative effects of smoking on mental health, we need to consider the impact of environmental, genetic, and other individual factors in relation to the effects of smoking and smoking cessation on mental health. By taking this individuality into account we will have a more comprehensive understanding of the association in question.

**Figure 1:**
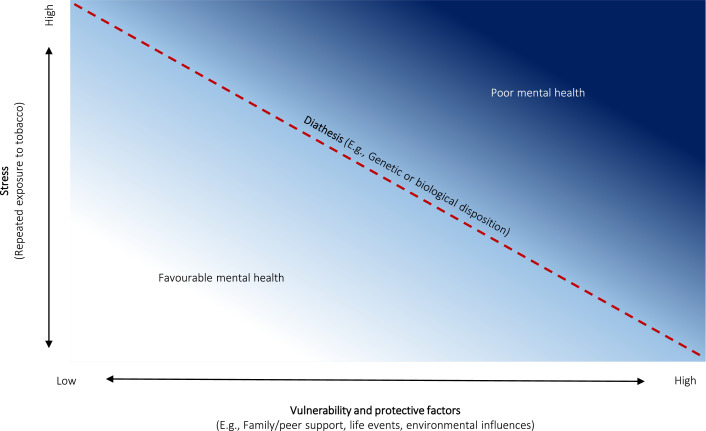
The stress diathesis model applied to smoking tobacco and mental health.

Stress-diathesis models are derived from the psychopathology literature and take into account individual variation in static and changeable factors (Slavik & Croake 2006), and may be a more appropriate way to examine the association between smoking and mental health. The model suggests that developing mental ill-health is heterogeneous in nature, with some people developing symptoms primarily because of genetic or biological factors and other individuals developing symptoms as the result of environmental factors or life events. However, the model also suggests that simplicity in aetiology is likely to be the exception rather than the rule; each of these factors interact with each other and their weighted contribution is different for each individual. Figure 1 displays the stress-diathesis model and its three main components: *stress*, which can be physical or emotional or another factor which causes negative effects on a person; *diathesis*, a genetic or biological disposition/threshold for development of mental ill-health; and *vulnerability or protective factors*, factors which help keep a person from developing mental ill-health or not, such as family or peer support, or other environmental influences.

In this model, smoking is the *stressor*. Smoking can be seen as a psychobiological stressor as it can damage the nervous system (Benowitz, 2010; Hallstrand et al., 2014; Salem et al., 2021) and there is evidence that neurological systems and oxidative stress pathways and inflammation markers return to normal functioning after sustained smoking cessation (Mamede et al., 2007; Salem et al., 2021). Thus, there is convincing evidence that smoking has a causal effect on mental health, but we suggest that the magnitude of this effect is moderated by the individuals’ diathesis to develop mental ill-health and their individual vulnerability and protective factors. Equally, the effect of smoking on mental health could be reversible, or partially reversible; however, the size of the improvement depends, again, upon the individual's diathesis, and vulnerability and protective characteristics.

If our model is valid, it suggests that people who are already at a high vulnerability and/or diathesis to develop mental ill-health could have their psychological state worsened through smoking. But moreover, these concepts function on a continuum, such that smoking (and higher levels of smoking) will move the smoker further along the spectrum towards being more mentally unwell. Second, the model accounts for elements of a common-cause hypothesis which suggests that environmental or genetic factors may play a part in the association between smoking, smoking cessation and mental health (Abdellaoui et al., 2021).

Data reported from recent adequately powered studies applying novel causal methods suggest that smoking cessation causally decreases the prescription of antidepressants and anxiolytics, and that smoking causally increases the risk of (symptoms of) depression, bipolar and schizophrenia disorder, with a weaker reverse association. There is however variation between effect sizes derived from these studies and based on the stress-diathesis model it is possible that the size of these effects is moderated by vulnerability or protective factors, and one's diathesis to develop mental ill-health.

### Implications of the model

Based on the current evidence for our stress-diathesis model, those delivering or receiving tobacco treatments can be reassured that on-average mental health will improve after breaking the tobacco withdrawal cycle, and at minimum smoking cessation will not harm mental health (Box 1). Future research should focus on: (1) identifying which traits mediate and moderate mental health outcomes in RCTs of smoking cessation interventions as these traits may be potential targets for intervention-optimisation, (2) determining the predictive strength of this model in different types of mental health disorders, (3) disentangling which constituents of tobacco are to blame for mental ill-health, and the implications that this has for the role that e-cigarettes may play in mental health, and (4) examining the role of exposure time on mental health, i.e., is there a dose-response effect of smoking cessation duration on mental health?

## Funding

GT is funded by a Cancer Research UK Population Researcher Postdoctoral Fellowship award (reference: C56067/A21330) and Cancer Research UK project award (reference: PPRCPJT\100,023). JT is funded by a Veni grant from the Netherlands Organization for Scientific Research (NWO, reference: 016.Veni.195.016).
